# Arrhythmias After Tetralogy of Fallot Repair

**Published:** 2005-10-01

**Authors:** Antonio Franco Folino, Luciano Daliento

**Affiliations:** Department of Cardiology, University of Padua, Italy

**Keywords:** Tetralogy of Fallot, surgery, ventricular arrhythmias, risk stratification

## Abstract

Tetralogy of Fallot is the most common cyanotic congenital heart disease, with a good outcome after total surgical correction. In spite of a low perioperative mortality and a good quality of life, late sudden death remains a significant clinical problem, mainly related to episodes of sustained ventricular tachycardia and ventricular fibrillation. Fibro-fatty substitution around infundibular resection, intraventricular septal scar, and patchy myocardial fibrosis, may provide anatomical substrates of abnormal depolarization and repolarization causing reentrant ventricular arrhythmias.

Several non-invasive indices based on classical examination such as ECG, signal-averaging ECG, and echocardiography have been proposed to identify patients at high risk of sudden death, with hopeful results. In the last years other more sophisticated invasive and non-invasive tools, such as heart rate variability, electroanatomic mapping and cardiac magnetic resonance added a relevant contribution to risk stratification.

Even if each method per se is affected by some limitations, a comprehensive multifactorial clinical and investigative examination can provide an accurate risk evaluation for every patient.

## Introduction

Since the first surgical correction was performed in 1954 [1], several epidemiologic studies evidenced the good prognosis of the patients operated on for total correction of tetralogy of Fallot (TOF), with a perioperative death less than 1% [2], and a survival rate 30 years after the surgery very close to 90% [3-8].

On the other hand, a more accurate analysis of survival curves shows as these patients are characterized by a relevant late mortality that increases 25 years postoperatively from 0.24%/yrs to 0.94%/yrs [9]. Its main cause is sudden cardiac death (35-45%) essentially due to episodes of sustained ventricular tachycardia or ventricular fibrillation.

All the pathophysiological mechanisms responsible for cardiac arrhythmias can be present in patients after repair of TOF. However, electrical instability results mainly from anatomical  modifications following surgery or from mechanical events such as ventricular dilation and stretching[10]. In particular, abnormal fibrous tissue was found at different sites in the right and left ventricles, as well as fibrous fatty substitution can be present around the surgical scar, and could represent an anatomical substrate for reentrant arrhythmias, as well evidenced by endocardial mapping [11,12]. These alterations also represent the  substrate for local abnormalities in ventricular depolarization and repolarization [13].

In the last 10 years the most challenging issue was to identify some risk factors able to identify patients prone to develop severe ventricular arrhythmias and sudden death, particularly by means of non- invasive techniques, and the several studies published on this topic provided hopeful results. However, arrhythmic risk stratification in patients frequently showing complex supraventricular or ventricular arrhythmias [14], remains a challenging clinical problem.

## Bradyarrhythmias

Following the improvement of the surgical procedure, the incidence of bradyarrhythmias after TOF repair has been drastically reduced. Only in the initial years after the introduction surgical repair, occurrence of complete AV block[15], or sinus node dysfunction[16], requiring pacemaker implantation, were reported in some cases.

The modality of the surgical approach in the genesis of the block seems not to be relevant, as right atriotomy has been showed to reduce the prevalence of right bundle branch block but not that of the AV block[17]. Rarely, a late appearance after the operation of a complete AV block is reported [18,19], but its manifestation represents one of the causes of late mortality. Other authors reported a recovery of complete AV block within one month, showing moreover, that those patients with a later recovery of the conduction disturbance were characterized by a higher incidence of sudden death and a lower long-term survival [20].

A more recent study [21], conducted on a large Japanense population, showed a prevalence of bradyarrhythmias of about 8%, with an incidence of 2nd and 3rd degree AV  block respectively around 4% and 3%. The large majority of patients with 3rd degree AV block were characterized by perimembranous ventricular septal defect. Moreover, it is noteworthy that in none of the patients who had perioperative complete AV block the conduction disturbance persisted later, and that one patient with complete AV block, who did not have pacemaker implantation, died during follow-up.

## Supraventricular Arrhythmias

The incidence of atrial arrhythmias after TOF repair is relatively high, about 30%, including atrial fibrillation, flutter, focal or reentrant atrial tachycardia [16].

In spite of the absence of acute consequences, occurrence of supraventricular tachyarrhythmias is one of the main cause of morbidity in adult patients after surgical repair of TOF [16], being associated with an increased incidence of heart failure, reoperation, ventricular tachycardia, stroke and death. Patients suffering from such arrhythmia are characterized by the presence of increased right atrial volumes, pulmonary regurgitation and older age at surgical repair [22].

The anatomical substrate for atrial tachycardia is more frequently represented by reentrant circuits that are often multiple and unstable, and for this reason successful catheter ablation is difficult to be achieved. In some patients the presence of macroreentrant circuits localized around the atriotomy scar, were clearly evidenced by means of electroanatomic mapping [23]

## Risk Stratification for Ventricular Arrhythmias and Sudden Death

Several studies were undertaken to identify the more reliable method to stratify arrhythmic risk after surgical repair of TOF. Even simple clinical characteristics, such as the age at operation were shown to be associated with a higher risk of atrial and ventricular tachyarrhythmias and sudden death [4,6,24], are able to provide relevant prognostic information, research was mainly referred to instrumental non-invasive methods.

### Electrocardiographic features

Intraventricular conduction is typically prolonged in patients after TOF repair, and characterized by the presence of a right bundle branch block. However, QRS duration was one of the first indices showing a significant correlation with the occurrence of ventricular arrhythmias [24-27]. Particularly, it is generally accepted that the patients with a QRS longer than 180 msec are at higher risk. A similar limit was found to be useful to identify subjects with inducible sustained monomorphic ventricular tachycardia during programmed stimulation, where a high specificity was found for QRS duration =180 msec [28]. Moreover, it was observed that a late and rapid increase in QRS duration after surgery, better characterized patients who developed ventricular tachycardia or died suddenly [24].

QRS lengthening after TOF repair is probably the result of a combined effect of the surgical injury on the myocardium and on right bundle branch [29], and of the right ventricular enlargement [26]. Therefore, we cannot consider a prolonged QRS the specific expression of a delayed intraventricular conduction from an arrhythmogenic substrate, but a non-specific marker of electrical instability.

Standard ECG also provided other helpful indexes for risk stratification. Besides QRS duration, QRS dispersion also is considered a useful marker to stratify patients at higher risk for life-threatening arrhythmias [27]. A different QRS duration among the 12 leads probably more clearly denotes a different conduction velocity between right and left ventricle, mirroring the presence of localized myocardial alterations.

We described in 1995  the clinical significance of temporal and spatial QT dispersion, assumed to reflect inhomogeneous ventricular repolarization, as independent predictive factors of ventricular instability in patients operated on for total correction of TOF by ventriculotomy with a cut-off of 80 and 65 ms respectively. QT dispersion was unrelated to the presence of right bundle branch block and to functional conditions of the right and left ventricles, particularly ventricular dilatation. It is probably related to myocardial substrates secondary to ventriculotomy. In fact, patients with uncorrected TOF and patients undergoing correction through a transatrial approach showed significantly lower QT dispersion values than patients whose correction included ventriculotomy. In our series at uni and multivariate analysis, QT dispersion and end-diastolic volume of the right ventricle were the parameters that discriminate between patients with and without ventricular tachycardia [30].

According to the amounts of QT dispersion and right ventricular end-diastolic volume, we can calculate the probabilities for sustained ventricular tachycardia. For example, with an end-diastolic volume of 100 ml/m^2^ the probability varies between 0 and 30% according to different values of QT dispersion. Other studies on intervals dispersion such as QT, QTc, JT, showed divergent results [25,27].

### Signal-averaged ECG

**Figure 1:**
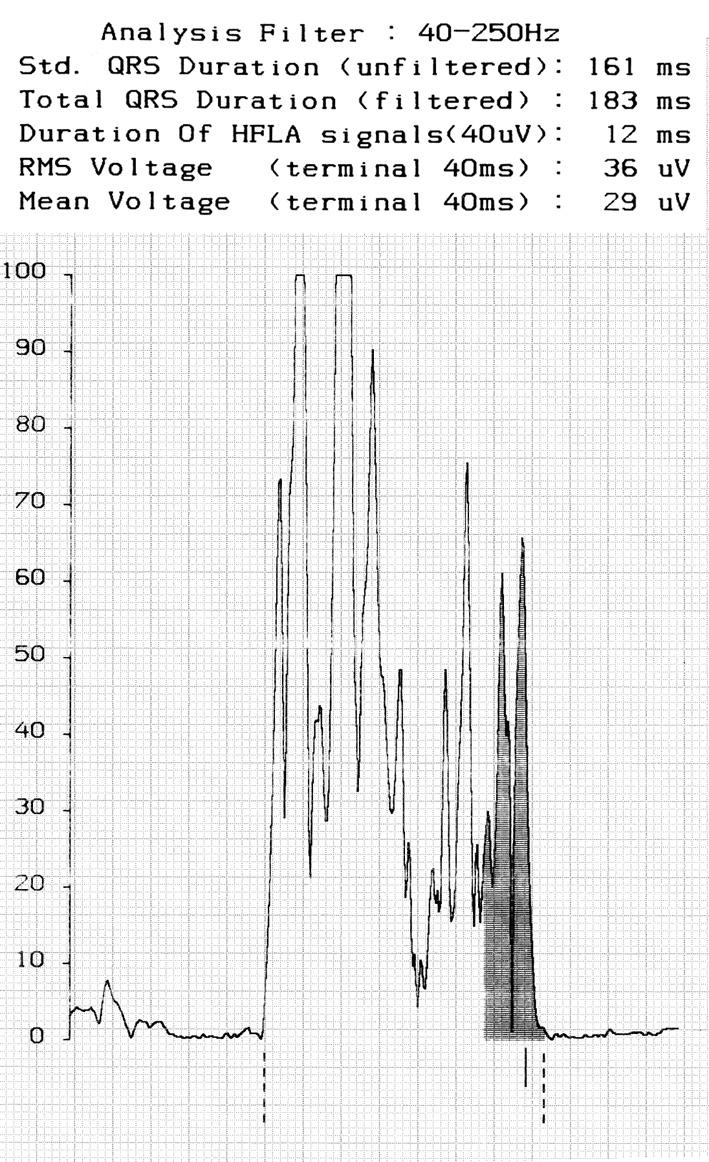
Signal-averaging ECG in a patient after repair of tetralogy of Fallot. The total duration of filtered QRS is 183msec, whereas a shorter unfiltered QRS is present (161msec). High frequency low amplitude signal are tightly represented (12ms), showing the absence of signals referred to typical late potentials

The use of a specific, more sophisticated analysis of ventricular depolarisation, by means of techniques such as signal-averaged ECG, provides a more accurate analysis of the signals that form the QRS complex, providing a better comprehension of the factors leading to QRS prolongation. The filtering procedure can provide a shorter or a longer QRS in comparison with standard measurement, magnifying the presence of specific components with high frequency and low amplitude characteristics, that more specifically can represent delayed intraventricular conduction disturbances, potentially responsible for reentrant ventricular arrhythmias. (Figure 1)

The use of this method evidenced a high prevalence of late potentials in patients after TOF repair, about 30%. However, analysis of the correlation between their presence and the occurrence of severe ventricular arrhythmias provided contrasting results [30-34]. The reasons for such discordance could be mainly related to the different technical instruments used and for the arbitrary classification of positive late potentials. However, we probably can conclude that signal-averaged ECG represents a useful method to identify patients with episodes of nonsustained ventricular tachycardia, but its usefulness in  predicting more severe ventricular arrhythmias and sudden death is uncertain.

### Holter monitoring

Ambulatory ECG monitoring is one of the more simple and accessible techniques to analyze the arrhythmic profile in subjects at higher risk for sudden death. In patients after TOF repair, isolated ventricular ectopic beats are frequently present during 24-hour recordings [14]. However, their presence rarely represents a predictor for life-threatening arrhythmias[35]. Also the presence of episodes of nonsustained ventricular tachycardia seems not related to an increased risk of more severe ventricular arrhythmias or sudden death [36]. On the other hand, the clinical usefulness of Holter monitoring could be improved by the use of the recording to analyze time domain heart rate variability [40]. Finally, we have to consider that the prognostic significance of detected arrhythmias can change during the follow-up and becomes life-threatening, particularly in the presence of poor ventricular function, significant residual structural abnormalities, and by modifications of autonomic activity.

### T wave alternans

First applied in patients with myocardial infarction, analysis of microvolt T wave alternans is considered a marker of increased risk for severe ventricular arrhythmias and sudden death. This technique was also adopted in patients after repair of TOF[37], showing a prevalence of T wave alternans similar of normal subjects, and without significant correlation with nonsustained ventricular tachycardia occurrence.

### Structural and hemodynamic abnormalities of the right ventricle

Echocardiographic examination provides relevant information that can be used in risk stratification. The presence of structural abnormalities of the right ventricle, such as outflow tract aneurysm, and pulmonary [24] or tricuspid regurgitation, were found more frequently in patients with episodes of sustained ventricular tachycardia[35]. Also the simple analysis of right ventricular volume represents a prognostic factor for episodes of sustained ventricular tachycardia or ventricular fibrillation[26,27]. Moreover, a higher ratio of right to left ventricular systolic pressure after surgery was shown as an independent predictor of long-term survival[6].

### Autonomic nervous system

**Figure 2:**
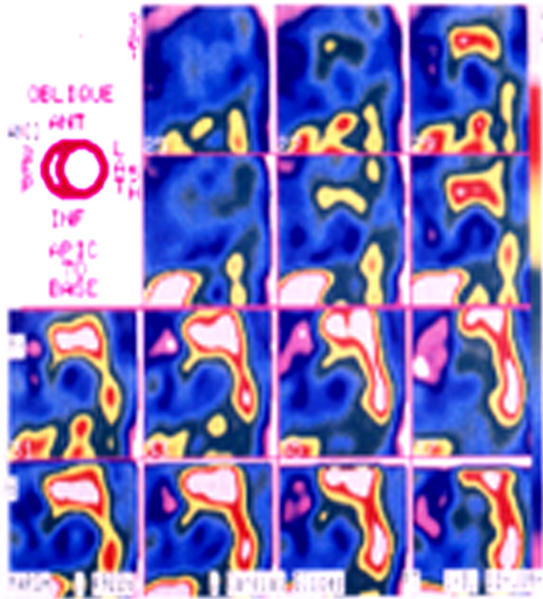
MIBG scintigraphy showing the presence of structural abnormalities involving nerve endings and receptors in a patients after tetralogy of Fallot repair. This findings characterize patients with severe ventricular arrhythmia

As in other cardiac diseases, autonomic nervous system plays a crucial role in the genesis of ventricular arrhythmias also in patients after surgical repair of TOF. In these subjects not only functional abnormalities induced by a reduced cardiac performance are present, but also structural abnormalities involving nerve endings and receptors, as evidenced by means of MIBG technique [38], particularly in subjects at higher risk for severe ventricular arrhythmias. Inhomogeneity of adrenergic fibres was more evident in patients with the highest ventricular end-diastolic volumes and the lowest ejection fraction. (Figure 2)

Alteration of autonomic activity was confirmed by different non-invasive methods, such as heart rate variability analysis and baroreflex sensitivity [39]. The presence of an unbalanced  autonomic activity, with a sympathetic dominance and a reduced vagal tone, is associated with an increased risk for severe ventricular arrhythmias and sudden death. Among the different indexes of heart rate variability, the standard deviation of RR intervals, evaluated over 24-hour period, seems to be the more reliable parameter to be considered [40].

### Electrophysiologic study

The role of electrophysiologic study in arrhythmic risk stratification has been debated for a long time. Different studies showed that patients with no inducible sustained ventricular tachycardia have a better prognosis [41,428]. However, the interpretation of test outcome is sometimes difficult due to a low specificity of the positive responses.

A recent study [43] conducted on 252 patients with repaired TOF, showed that programmed ventricular stimulation, with inducible monomorphic or polymorphic ventricular tachycardia, predicted the occurrence of spontaneous episodes of ventricular tachycardia and sudden death during a long-term follow-up.

**Figure 3:**
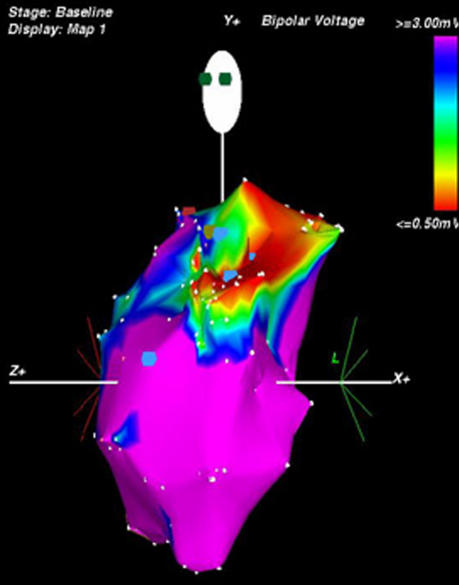
Three-dimensional electroanatomic voltage mapping (CARTO) of the right ventricle, showing an inhomogeneous activation pattern, particularly in the outflow tract (upper part of the map), providing a clear representation of a potential  reentry circuit responsible for ventricular tachycardia

In the last few years, the use of electroanatomic voltage mapping of the right ventricle outflow tract, provided a clear representation of the reentry circuit responsible for ventricular tachycardia, increasing diagnostic accuracy and helping in transcateter ablation of the circuit. (Figure 3).

### Magnetic resonance

Cardiac magnetic resonance imaging (MRI) represents a new technique, useful in the evaluation of ventricular volumes and in the quantification of valvular regurgitation [44]. Thus, MRI is a helpful technique in assessment of postoperative patients, especially in disorders in which the right ventricle has been affected. Moreover, it can characterize cardiac tissue if associated with the use of gadolinium.

**Figure 4:**
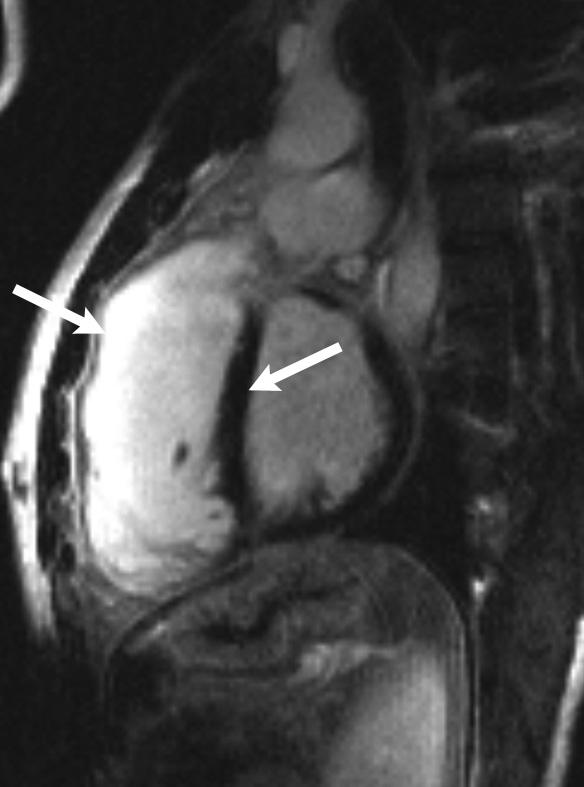
Cardiac magnetic resonance with delayed contrast enhancement in a patient after tetralogy of Fallot repair. The areas with fibrosis have an increased signal intensity (white areas, left arrow) as compared with those of healthy myocardium (dark areas, right arrow)

Current cardiac MRI techniques, by means of the protocols based on delayed contrast enhancement, first applied to patients with previous myocardial infarction [45,46], provide precise delimitation of areas of myocardial  necrosis or fibrosis. On delayed contrast-enhanced images, the areas of fibrosis have an increased signal intensity (white areas) as compared with those of healthy myocardium (dark areas). (Figure 4).

The detection of late-enhancement in patients operated on for TOF, reflects the presence of fibrotic tissue around the scar and the insertion of infundibular and interventricular patches. According to our preliminary data, we demonstrated the presence of late-enhancement around infundibular patch and right ventricular anterior wall in a very large proportion of patients with severe ventricular arrhythmias and abortive sudden death, when compared with those without arrhythmias. Moreover, we found a concordance between the presence and location of right ventricular low voltage areas identified by the electro-anatomical mapping and the presence of increased late enhancement described at MRI. The low voltage areas were recorded predominantly around the infundibular patch region and in the right ventricular anterior wall, the same distribution of the increased late enhancement revealed with MRI. In patients operated on for TOF, these areas represent regions affected by a surgical damage due to infundibulotomy (the enlargement of the infundibular region by means of a patch) and ventriculotomy, with the consequent formation of myocardial atrophy, myocyte loss and fibro-fatty replacement. The co-existing scar and viable myocardium create opportunities for electrical reentry.

## Treatment of Arrhythmias

Large randomized and controlled trials for specific drug treatment are lacking in TOF. The reasons are probably related to the low number of patients that need to be treated for prevention of sudden death. Different antiarrhythmic drugs were proposed, with successful results [47], however, we can consider that the presence of ventricular beats or non-sustained ventricular tachycardia are not markers of higher risk for sudden death, and does not always require a specific pharmacological treatment.  Furthermore, the arrhythmic risk may be increased by the administration of potentially proarrhythmic drugs, as well as side effects due to their chronic use have to be considered.

The prevention of sudden death by antiarrhythmic drugs should be restricted to patients with complex ventricular arrhythmias or sustained ventricular tachycardia, in the absence of severe symptoms or residual hemodynamic substrates amenable to surgical reintervention such as residual pulmonary stenosis or pulmonary valve incompetence.

ICD implantation can be a useful option to prevent life threatening arrhythmias[48]; however, its use should be limited to patients with severe right ventricular dysfunction who have been resuscitated after ventricular fibrillation or who have had episodes of syncope with rapid sustained ventricular tachycardia in spite of chronic use of amiodarone alone or associated with ß-blockers.

Some authors proposed an intraoperative treatment for ventricular tachycardia, by means of surgical resection and cryoablation, following endocardial mapping [11,12,49], with good results. The successful use of transcatheter radiofrequency ablation is documented in some cases as well [50][51]. Similarly, ablation of macro-reentrant circuit responsible for right atrial tachycardia showed promising results [23]. However, the major limitation for all the techniques aiming to interrupt a reentrant pathway is that the presence of multiple circuits is possible in these patients, fdue to the complexity of anatomical alteration, particularly in the vicinity of surgical scar.
